# Association between long noncoding RNA rs944289 and rs7990916 polymorphisms and the risk of colorectal cancer in a Chinese population

**DOI:** 10.1038/s41598-022-06474-3

**Published:** 2022-02-15

**Authors:** Yan Wang, Zhiyuan Qiu, Guangyu Tian, Qianqian Zhu, Zhao Zhang, Rong Qin, Yong Peng, Weifeng Tang, Sheng Zhang, Yan Xi

**Affiliations:** 1 Department of Oncology, Affiliated People’s Hospital of Jiangsu University, Zhenjiang, Jiangsu China; 2 Department of Geriatrics, Affiliated People’s Hospital of Jiangsu University, Zhenjiang, Jiangsu China; 3 Department of Oncology, People’s Hospital of Jiangdu, Yangzhou, Jiangsu China; 4 Department of Cardiothoracic Surgery, Nanjing Drum Tower Hospital, Nanjing, Jiangsu China; 5 Department of General Surgery, Changzhou No. 3 People’s Hospital, Changzhou, Jiangsu China

**Keywords:** Cancer, Gastrointestinal cancer

## Abstract

Long non-coding RNAs (LncRNAs) play vital roles in the tumorigenesis of many cancers. Single nucleotide polymorphisms (SNPs) of the lncRNA also play vital roles in tumorigenesis. We explored lncRNA rs944289 and rs7990916 polymorphisms and analyzed the relationship between these lncRNA polymorphisms with the colorectal cancer (CRC) risk in a Chinese population. We recruited 1003 CRC patients from the Affiliated People’s Hospital of Jiangsu University and the Fujian Medical University Union Hospital from October 2014 to August 2017. Genomic DNA was extracted using a DNA Kit from lymphocytes of peripheral blood and the genotyping was performed with a SNPscan method. We found that the rs944289 TT homozygote was associated with the decreased CRC risk in the overall population. LncRNA rs944289 TT decreased the CRC risk in the subgroup of female, male, age ≥ 61, without alcohol intake, smoking and BMI ≥ 24 by logistic regression. The subgroup analysis revealed that lncRNA rs7990916 was not associated with CRC risk except for age < 61. Logistic regression analysis revealed that lncRNA rs944289 TT homozygote was associated with the increased risk of rectum cancer (TT vs. CC + CT: adjusted OR = 1.29, 95% CI 1.10–1.66, *P* = 0.041) or colon cancer. In summary, we proved that lncRNA rs944289 might be significantly related to the decreased CRC risk in the Chinese Han populations and lncRNA rs7990916 was not associated with the CRC risk except for patients of age < 61. In the future, studies with larger samples should be conducted to validate our results.

## Introduction

Colorectal cancer (CRC) has ranked third in terms of incidence but second in terms of mortality in the world^1^. In China, CRC has ranked both fifth in terms of incidence and mortality^2^. The incidence of CRC has been increasing, mostly due to unhealthy lifestyle, aging and environmental factors^3^, but accumulating evidences have shown that an individual’s inherited factors also contribute to the development of CRC.

Long noncoding RNAs (lncRNAs) are a sort of noncoding RNA molecules with more than 200 nucleotides in length, and are disease- or tissue-specific expression patterns^4,5^. LncRNAs play crucial roles in chromatin dynamics, genome packaging, gene regulation, cellular pathways and biological processes^6^. LncRNAs also play viotal roles in the oncogenesis and metastasis of CRC^7,8^. Yu et al. found that linc-UFC1 participated in the progression of CRC, and overexpression of linc-UFC1 in CRC patients was positively associated with tumor grade and stage^7^. Zhao et al. also reported overexpression of Linc-A was correlated with poor survival in CRC patients^8^.

Single nucleotide polymorphisms (SNPs) occurring in the functional region of lncRNAs can influence disease risk and can also promote cancer development^9^. LncRNA rs944289 and rs7990916 polymorphisms increased the risk of different diseases. For example, lncRNAs rs7990916 TT genotype was found to increase Alzheimer’s disease (AD) risk^10^. Papillary thyroid carcinoma susceptibility candidate 3 (PTCSC3) rs944289 polymorphism increased the risk of papillary thyroid cancer (PTC)^11,12^. Cao et al. also reported that lncRNA rs944289 may increase the risk of esophagogastric junction adenocarcinoma (EGJA)^13^. However, Xu et al*.* found that lncRNA rs944289 have no effect on the risk of breast cancer (BC)^14^. These data indicate SNPs in lncRNAs play important roles in tumorigenesis. Although lncRNA rs944289 and rs7990916 polymorphisms have a risk effect on some diseases, there are no studies concerning the relationship of these two variants with CRC risk. Based on previous studies, we explored lncRNA rs944289 and rs7990916 polymorphisms in CRC patients and analyzed the relationship between these lncRNA polymorphisms and CRC risk.

## Materials and methods

### Patients and samples

We recruited 1003 CRC patients from the Affiliated People’s Hospital of Jiangsu University and the Fujian Medical University Union Hospital from October 2014 to August 2017. Patients with histologically confirmed CRC were enrolled and the major exclusion criteria were as follows: (1) presence of immunological diseases, (2) with other primary cancers, and (3) with other colonic diseases (e.g. Crohn’s disease), (4) exposure to antitumor treatments before surgery. One thousand three hundred and three healthy subjects were selected as healthy controls from the departments of physical examination in these two hospitals. The healthy controls were Chinese Han populations with the following exclusion criteria: (1) with history of any cancer, (2) with metabolic or autoimmune diseases, and (3) with liver/kidney dysfunction, (4) with any other systemic diseases. The demographic characteristics were recorded, including body mass index (BMI), age, gender, alcohol intake and smoking.

The study was approved by the Ethics Committee of the Affiliated People's Hospital of Jiangsu University and conducted according to the Declaration of Helsinki. All participants signed an informed consent before enrolled in this research and all methods were performed in accordance with the relevant guidelines and regulations.

### SNP genotyping

We collected blood samples from each participant after their admission to the hospital. The blood samples collected in a test tube containing EDTA were used for genotyping assay. Genomic DNA was extracted using a DNA Kit (Promega, Madison, USA) from lymphocytes of peripheral blood. The DNA was quantified by a measurement of OD260 and then was stored at -80℃. SNPs of lncRNA rs944289 and rs7990916 were analyzed by a SNPscan Kit (Genesky Biotechnologies Inc., Shanghai, China)^15^. 4% DNA samples were selected randomly and analyzed by PCR/Sanger sequencing. The rs944289 locus primers were: 5-TGGTTGAAAGATAGTCATTG-3 (forward) and 5-AGATTTGTAATAGCTGGGAA-3(reverse). The rs7990916 locus primers were: 5-CTTTGTATCCTTCATTCTTA-3 (forward) and 5-CAAGTTGACACTCAGAATTA-3(reverse). For quality control, replicate blinded samples were included to check for the reproducibility of the results by another technician and the results were unchanged.

### Statistical analysis

All data were analyzed by using SPSS 23.0 (IBM Corporation, Armonk, NY, USA). The chi-square or Fisher’s exact test was applied to compare the categorical variables, whereas the continuous variables were evaluated by the student’s t-test or Wilcoxon signed-rank test. The relationship between the lncRNA rs944289/7990916 polymorphism and CRC risk was assessed by odds ratios (OR) and 95% confidence intervals (CIs). Logistic regression model was conducted to analyze the associations among lncRNA rs944289 and rs7990916 polymorphisms, the clinical characteristics and CRC risk. Hardy–Weinberg equilibrium (HWE) was used to analyze the genotype distributions of lncRNA rs944289/7990916. A *P* < 0.05 (two-tailed) was adopted as the statistically significant level.

### Ethics approval and consent to participate

The Ethics Committee of the Affiliated People’s Hospital of Jiangsu University approved the protocol of the study (K-20210105-W), and all participants signed an informed consent before enrolled in this research.

### Consent for publication

The authors appreciate all the patients in this work for their cooperation and permission for the publication of the article.

## Results

### Characteristics of CRC patients

In our study, 1003 CRC cases (431 colon cancer and 572 rectum cancer patients) and 1303 healthy controls were enrolled to investigate the correlation of the two SNPs (rs944289 and rs7990916) with CRC risk. Table 1 listed detailed demographics data. The mean age of CRC patients and controls were 61.10 ± 12.17 years and 61.40 ± 9.61 years, respectively. There were no statistically significant differences in age and gender between CRC patients and controls (both *P* > 0.05). However, smoking, alcohol intake and BMI increased the risk of CRC (both *P* < 0.05), so we adjusted these factors by multiple logistic regression analyses.

**Table 1:** The demographic variables of CRC patients and controls.

Variable	Cases (n = 1003)		Controls (n = 1303)		*P*^a^
	n	%	n	%	
Age (years)					0.61
< 61	451	44.97	600	46.05	
≥ 61	552	55.03	703	53.95	
Sex					0.87
Male	620	61.81	801	61.47	
Female	383	38.19	502	38.53	
BMI (kg/m^2^)					**< 0.001**
< 24	670	66.80	688	52.80	
≥ 24	333	33.20	615	47.20	
Alcohol intake					**< 0.001**
Never	829	82.65	1167	89.56	
Ever	174	17.35	136	10.44	
Smoking					**0.002**
Never	744	74.18	1038	79.66	
Ever	259	25.82	265	20.34	
Site of tumor					
Colon cancer	431	42.97			
Rectum cancer	572	57.03			

BMI, body mass index.

^a^Bold values are statistically significant (*P* < 0.05).

### Primary information for lncRNA rs944289 and rs7990916

The genotypic frequencies of lncRNA rs944289 and rs7990916 met the HWE (*P* = 0.105 and *P* = 0.359, respectively). Minor allele frequency (MAF) of lncRNA rs944289 polymorphism was 0.28, which was similar to SNP database for Chinese populations (MAF = 0.24). MAF of lncRNA rs7990916 polymorphism was 0.23, which was similar to SNP database for Chinese populations. Table 2 summarized the corresponding information of lncRNA rs944289 and rs7990916.

**Table 2:** Primary information for LncRNA rs 944289 C>T and rs7990916 C>T polymorphisms.

Genotyped SNPs	Chromosome	Chr Pos (NCBI Build 38)	Region	MAF^a^ for Chinese in database	MAF in our controls (n = 1303)	*P* value for HWE^b^ test in our controls	Genotyping value (%)
rs944289 C>T	14	36180040	nc transcript variant	0.24	0.28	0.11	98.66
rs7990916 C>T	13	80065389	nc transcript variant	0.21	0.23	0.36	98.87

^a^MAF: minor allele frequency;

^b^HWE: Hardy–Weinberg equilibrium.

### Association of lncRNA polymorphisms with CRC risk in the overall population

The genotypes and allele distributions of lncRNA rs944289 and rs7990916 in CRC patients and controls were presented in Table 3. LncRNA rs944289 frequencies were 29.55% (CC), 46.73% (CT) and 23.72% (TT) in CRC patients, whereas in controls, the distributions of those genotypes were 29.69%, 51.62% and 18.69%, respectively. We found that rs944289 TT homozygote was associated with decreased CRC risk when compared with the CC or CC + CT (TT vs. CC: adjusted OR = 0.88, 95% CI 0.78–0.99, *P* = 0.037; TT vs. CC + CT: adjusted OR = 0.86, 95% CI 0.78–0.95, *P* = 0.004). LncRNA rs7990916 frequencies were 79.69% (CC), 19.49% (CT) and 0.82% (TT) in CRC patients, and 77.31% (CC), 20.92% (CT) and 1.77% (TT) in controls. There was no significant difference between lncRNA rs7990916 and the risk of CRC (all *P* > 0.05) in the overall population.

**Table 3:** Genotype Frequencies of LncRNA rs944289 C>T and rs7990916 C>T Polymorphisms and the CRC Risk.

Genotype	CRC Cases (n = 1003)		Controls (n = 1303)		Crude OR (95%CI)	*P*	Adjusted OR^a^ (95%CI)	*P*
	n	%	n	%				
**rs944289 C>T**								
CC	289	29.55	386	29.69	1.00		1.00	
CT	457	46.73	671	51.62	0.91 (0.75–1.10)	0.34	1.07 (0.88–1.30)	0.53
TT	232	23.72	243	18.69	1.27 (1.01–1.61)	**0.043**	0.88 (0.78–0.99)	**0.037**
CT + TT	689	70.45	914	70.31	1.01 (0.84–1.21)	0.94	0.97 (0.81–1.17)	0.75
CC + CT	746	76.28	1057	81.31	1.00		1.00	
TT	232	23.72	243	18.69	1.35 (1.10–1.66)	**0.004**	0.86 (0.78–0.95)	**0.004**
T allele	921	47.09	1157	44.50				
**rs7990916 C>T**								
CC	781	79.69	1005	77.31	1.00		1.00	
CT	191	19.49	272	20.92	0.90 (0.73–1.14)	0.34	0.89 (0.72–1.10)	0.27
TT	8	0.82	23	1.77	0.45 (0.20–1.01)	0.066	0.45 (0.20–1.03)	0.059
CT + TT	199	20.31	295	22.69	0.87 (0.71–1.06)	0.18	0.85 (0.70–1.05)	0.13
CC + CT	972	99.18	1277	98.23	1.00		1.00	
TT	8	0.82	23	1.77	0.46 (0.20–1.03)	0.067	0.47 (0.21–1.06)	0.067
T allele	207	10.56	318	12.23				

Bold values are statistically significant (*P* < 0.05).

^a^Adjusted for age, gender, smoking, alcohol use and BMI status.

### Stratified analyses between lncRNA rs944289 polymorphism and CRC risk

Stratified analysis was performed and revealed that rs944289 TT genotype was associated with the decreased CRC risk in the subgroup of male (adjusted OR = 0.88, 95% CI 0.77–0.99, *P* = 0.045), female (adjusted OR = 0.81, 95% CI 0.68–0.99, *P* = 0.097), age ≥ 61 (adjusted OR = 0.86, 95% CI 0.74–0.99, *P* = 0.035), smoking (adjusted OR = 0.66, 95% CI 0.53–0.81, *P* < 0.001), never alcohol intake (adjusted OR = 0.88, 95% CI 0.78–0.98, *P* = 0.021) and BMI ≥ 24 (adjusted OR = 0.82, 95% CI 0.696–0.94, *P* = 0.0016) (Table 4).

**Table 4:** Stratified analyses between LincRNA rs944289 C>T polymorphism and CRC risk by gender, age, BMI, smoking and alcohol intake.

Variable	LincRNA rs944289 C>T (case/control) ^a^			Adjusted OR ^b^ (95% CI); *P*				
	CC	CT	TT	CC	CT	TT	CT /TT	TT vs. (CT/CC)
**Sex**								
Male	176/231	276/407	150/161	1.00	1.08 (0.84–1.39); *P*: 0.56	0.91 (0.78–1.05); *P*: 0.20	0.99 (0.78–1.26); *P:* 0.94	0.88 (0.77–0.99); ***P*: 0.045**
Female	113/155	181/264	82/82	1.00	1.04 (0.76–1.43); *P*: 0.79	0.82 (0.67–1.00); *P*: 0.053	0.93 (0.69–1.25); *P*: 0.62	0.81 (0.68–0.97); ***P*: 0.019**
**Age**								
< 61	115/168	216/312	111/118	1.00	0.96 (0.71–1.30); *P*: 0.80	0.87 (0.72–1.03); *P*: 0.13	0.89 (0.67–1.19); *P*: 0.43	0.87 (0.75–1.02); *P*: 0.082
≥ 61	174/218	241/359	121/125	1.00	1.15 (0.88–1.49); *P*: 0.30	0.90 (0.77–1.06); *P*: 0.22	1.04 (0.81–1.33); *P*: 0.76	0.86 (0.74–0.99); ***P*: 0.035**
**Smoking**								
Never	223/314	351/520	154/201	1.00	1.02 (0.82–1.27); *P*: 0.88	0.93 (0.81–1.06); *P*: 0.28	0.91 (0.71–1.78); *P*: 0.49	0.93 (0.83–1.05); *P*: 0.25
Ever	66/72	106/151	78/42	1.00	1.26 (0.82–1.93); *P*: 0.29	0.70 (0.54–0.90); ***P*: 0.006**	0.95 (0.64–1.41) *P*: 0.79	0.66 (0.53–0.81); ***P*: 0.000**
**Alcohol intake**								
Never	238/348	387/598	184/218	1.00	1.03 (0.83–1.27); *P*: 0.81	0.88 (0.77–1.00); *P*: 0.056	0.94 (0.77–1.15); *P*: 0.56	0.88 (0.78–0.98); ***P*: 0.021**
Ever	51/38	70/73	48/25	1.00	1.33 (0.77–2.30); *P*: 0.30	0.86 (0.61–1.20); *P*: 0.38	1.14 (0.69–0.91); *P*: 0.61	0.77 (0.58–1.03); *P*: 0.074
**BMI (kg/m**^**2**^)								
< 24	202/216	303/341	150/129	1.00	1.05 (0.82–1.34); *P*: 0.71	0.90 (0.77–1.05); *P*: 0.17	0.97 (0.77–1.23); *P*: 0.80	0.88 (0.77–1.01); *P*: 0.064
≥ 24	87/170	154/330	82/114	1.00	1.10 (0.80–1.52); *P*: 0.56	0.84 (0.69–1.02); *P*: 0.085	0.97 (0.72–1.32); *P*: 0.86	0.82 (0.696–0.94); ***P*: 0.016**

^a^For ICAM-1 rs944289 C>T, the genotyping was successful in 980 (97.71%) CRC cases, and 1300 (99.77%) controls;

^b^Adjusted for multiple comparisons [age, gender, BMI, smoking status and alcohol intake (besides stratified factors accordingly)] in a logistic regression model.

### Stratified analyses between lncRNA rs7990916 polymorphism and CRC risk

We further analyzed stratified effects of lncRNA rs7990916 on CRC risk by logistic regression model. Genotype distributions of rs7990916 were evaluated with age, sex, BMI, drinking and smoking. Results show that there was no significant association between CRC patients and controls except for age < 61 (CT vs. CC: adjusted OR = 0.67, 95% CI 0.48–0.93, *P* = 0.018) (Table 5).

**Table 5:** Stratified analyses between LincRNA rs7990916 C>T Polymorphism and CRC risk by gender, age, BMI, smoking status and alcohol intake.

Variable	LincRNA rs7990916 C>T Polymorphism (case/control) ^a^			Adjusted OR ^b^ (95% CI); *P*				
	CC	CT	TT	CC	CT	TT	CT /TT	TT vs. (CT/CC)
**Sex**								
Male	477/619	123/166	4/14	1.00	0.94 (0.72–1.22); *P*: 0.63	0.382 (0.12–1.19); *P*: 0.096	0.90 (0.69–1.16); *P*: 0.40	0.39 (0.13–1.20); *P*: 0.099
Female	304/386	68/106	4/9	1.00	0.83 (0.59–1.18); *P*: 0.30	0.54 (0.16–1.79); *P*: 0.31	0.81 (0.58–1.13); *P*: 0.22	0.56 (0.17–1.87); *P*: 0.35
**Age**								
< 61	370/460	69/127	4/11	1.00	**0.67 (0.48–0.93); *P*: 0.018**	0.48 (0.15–1.53); *P*: 0.22	**0.66 (0.47–0.90); *P*: 0.010**	0.51 (0.16–1.65); *P*: 0.26
≥ 61	411/545	122/145	4/12	1.00	1.08 (0.82–1.43); *P*: 0.57	0.43 (0.13–1.37); *P*: 0.16	1.04 (0.79–1.36); *P*: 0.80	0.42 (0.13–1.34); *P*: 0.14
**Smoking status**								
Never	585/802	136/216	7/17	1.00	0.87 (0.68–1.10); *P*: 0.24	0.55 (0.22–1.36); *P*: 0.20	0.84 (0.66–1.07); *P*: 0.16	0.57 (0.23–1.40); *P*: 0.22
Ever	196/203	55/56	1/6	1.00	0.98 (0.64–1.51); *P*: 0.94	0.19 (0.02–1.58); *P*: 0.12	0.91 (0.60–1.38); *P*: 0.66	0.18 (0.02–1.57); *P*: 0.12
**Alcohol intake**								
Never	643/900	160/243	7/21	1.00	0.90 (0.71–1.12); *P*: 0.34	0.47 (0.20–1.11); *P*: 0.085	0.862 (0.69–1.08); *P*: 0.19	0.48 (0.20–1.14); *P*: 0.096
Ever	138/105	31/29	1/2	1.00	0.77 (0.44–1.38); *P*: 0.38	0.39 (0.04–4.44); *P*: 0.45	0.75 (0.43–1.32); *P*: 0.32	0.41 (0.04–4.54); *P*: 0.46
**BMI (kg/m**^**2**^)								
< 24	521/521	130/153	5/12	1.00	0.86 (0.66–1.12); *P*: 0.26	0.42 (0.14–1.19); *P*: 0.10	0.83 (0.64–1.07); *P*: 0.15	0.43 (0.15–1.23); *P*: 0.12
≥ 24	260/484	61/119	3/11	1.00	0.96 (0.66–1.35); *P*: 0.79	0.51 (0.14–1.87); *P*: 0.31	0.92 (0.66–1.29); *P*: 0.62	0.51 (0.14–1.87); *P*: 0.31

^a^For LincRNA rs7990916 C>T, the genotyping was successful in 977 (97.41%) CRC cases, and 1298 (99.62%) controls;

^b^Adjusted for multiple comparisons [age, sex, BMI, smoking status and alcohol intake (besides stratified factors accordingly)] in a logistic regression model.

Significant values are in [bold].

### LncRNA rs944289/rs7990916 polymorphism and CRC risk by site of tumor

Logistic regression analysis revealed that lncRNA rs944289 was associated with increased risk of colon cancer (TT vs. CC + CT: adjusted OR = 1.44, 95% CI 1.11–1.88, *P* = 0.007) or rectum cancer (TT vs. CC + CT: adjusted OR = 1.29, 95% CI 1.10–1.66, *P* = 0.041) when compared with CC + CT (Table 6). We also found lncRNA rs7990916 did not alter the risk of colon cancer (CT vs. CC: adjusted *P* = 0.18, TT vs. CC: adjusted *P* = 0.21, CT + TT vs.CC: adjusted *P* = 0.11, and TT vs. CC + CT: adjusted *P* = 0.24) or rectal cancer (CT vs. CC: adjusted *P* = 0.58, TT vs. CC: adjusted *P* = 0.11, CT + TT vs. CC: adjusted *P* = 0.37, and TT vs. CC + CT: adjusted *P* = 0.12) (Table 6).

**Table 6:** Stratified analyses between LncRNAs rs944289 C>T and rs7990916 C>T polymorphisms and CRC risk by site of tumor.

Genotype	Controls (n = 1303)		Colon cancer cases (n = 431)		Crude OR (95%CI)	*P*	Adjusted OR ^a^ (95%CI)	*P* ^a^	Rectum cancer cases (n = 572)		Crude OR (95%CI)	*P*	Adjusted OR ^a^ (95%CI)	*P* ^a^
	n	%	n	%					n	%				
**rs944289 C>T**														
CC	386	29.69	130	30.73	1.00		1.00		159	28.65	1.00		1.00	
CT	671	51.62	189	44.68	0.84 (0.65–1.08)	0.19	0.86 (0.67–1.12)	0.27	268	48.29	1.04 (0.82–1.30)	0.81	1.00 (0.79–1.27)	0.99
TT	243	18.69	104	24.59	1.27 (0.94–1.72)	0.14	1.33 (0.97–1.81)	0.073	128	23.06	1.28 (0.96–1.70)	0.10	1.27 (0.95–1.71)	0.10
CT + TT	914	70.31	293	69.27	0.95 (0.75–1.21)	0.71	0.97 (0.77–1.24)	0.84	396	71.35	1.05 (0.85–1.31)	0.70	1.07 (0.86–1.34)	0.53
CC + CT	1057	81.31	319	75.41	1.00		1.00		427	76.94	1.00		1.00	
TT	243	18.69	104	24.59	**1.42 (0.41–1.24)**	**0.010**	**1.44 (1.11–1.88)**	**0.007**	128	23.06	**1.35 (1.06–1.73)**	**0.015**	**1.29 (1.10–1.66)**	**0.041**
T allele	1157	44.50	397	46.93					524	47.21				
**rs7990916 C>T**														
CC	1005	77.31	342	80.85	1.00		1.00		439	78.81	1.00		1.00	
CT	272	20.92	77	18.20	0.83 (0.63–1.10)	0.21	0.82 (0.62–1.09)	0.18	114	20.47	0.96 (0.75–1.23)	0.76	0.931 (0.73–1.20)	0.58
TT	23	1.77	4	9.46	0.51 (0.18–1.49)	0.27	0.50 (0.17–1.48)	0.21	4	0.72	0.40 (0.14–1.16)	0.092	0.42 (0.14–1.22)	0.11
CT + TT	295	22.69	81	19.15	0.81 (0.61–1.06)	0.14	0.80 (0.88–1.36)	0.11	118	21.18	0.92 (0.72–1.17)	0.50	0.89 (0.70–1.14)	0.37
CC + CT	1277	98.23	419	99.05	1.00		1.00		553	99.28	1.00		1.00	
TT	23	1.77	4	9.46	0.53 (0.18–1.54)	0.37	0.52 (0.18–1.53)	0.24	4	0.72	0.40 (0.14–1.17)	0.093	0.42 (0.14–1.24)	0.12
T allele	318	12.23	85	10.05					222	19.93				

^a^Adjusted for age, sex, smoking status, alcohol use and BMI status.

Significant values are in [bold].

## Discussion

LncRNAs play an essential role in various biological processes, including cell proliferation, apoptosis, genomic imprinting, transcriptional interference and other critical processes^16,17^. Furthermore, lncRNAs have been reported to participate in the process of tumorigenesis in CRC. For instance, lncRNA CCAL could activate Wnt/β-catenin signaling pathway and induce multidrug resistance in CRC^18^. LncRNA SPRY4-IT1 could promote invasion and proliferation as a ceRNA of miRNA-101-3p in CRC^19^. Recently, numerous investigations have suggested lncRNA polymorphisms as one of the contributors to CRC risk. For example, Yang et al. reported lncRNA PCAT1 rs2632159 polymorphism increased CRC risk in a Chinese population^20^. Wang et al*.* also reported H19 rs2839698 polymorphism was associated with increased CRC risk^21^. On the other hand, lncRNA PRNCR1 rs13252298/rs1456315 and MALAT1 rs1194338 polymorphisms decreased the CRC risk^22,23^. Considering this background, we performed a case–control study to determine the relationship between lncRNA rs944289/rs7990916 polymorphism and CRC risk.

In this study, we found that lncRNA rs944289 TT homozygote could decrease CRC risk. After adjustment by multiple logistic regression, the TT genotype of lncRNA rs944289 was associated with the decreased CRC risk in the subgroups of female, male, age ≥ 61, BMI ≥ 24, smoking and never alcohol intake populations. LncRNA rs944289 have been confirmed to increase the risk of PTC^11,24^, and rs944289 predisposes to PTC by inhibiting the expression of PTCSC3^11,12^. PTCSC3 is a large intergenic noncoding RNA gene that is involved in the regulation of tumorigenesis. The SNP rs944289 is located 3.2 kb upstream of PTCSC3 and suppresses PTCSC3 by destroying a transcription factor-binding site in the promoter of PTCSC3^25,26^. Cao et al. also found that lncRNA rs944289 may increase the risk of EGJA in the smoking and age < 60 years populations^13^. However, in a Chinese population, Xu et al. discovered that lncRNA rs944289 have no significant effect on the risk of BC, which might be due to the specific tumorigenesis of BC^14^. However, up to now, no study has reported the association between lncRNA rs944289 polymorphism and CRC risk, and in this study, we observed significant relationships between the lncRNA rs944289 polymorphism and CRC risk.

Our results also revealed that there was no significant difference between lncRNA rs7990916 polymorphism and the CRC risk in an overall comparison. But when stratified by age < 61 years, lncRNA rs7990916 polymorphism decreased the risk of CRC. Similar to our results, Cao et al. observed no close relationship between lncRNA rs7990916 polymorphism and EGJA patients. In contrast, Jendrzejewski et al. found significant relationship between lncRNA rs7990916 and the risk of AD in the Europe populations. But the etiology of CRC is primarily different from that of AD^21^. Thus, these distinct conclusions on the lncRNA rs7990916 polymorphism may be mainly attributed to the heterogeneity of ethnics and disease types.

Nevertheless, the mechanism of lncRNA polymorphisms on the tumorigenesis of CRC is unclear. Previous studies reported that lncRNA GAS5 rs55829688 polymorphism increased CRC risk by modulating the binding affinity of the transcription factors YY1 to GAS5 promotor^27^. Moreover, MALAT1 rs664589 polymorphism increased CRC risk by binding to miRNA-194-5p, which resulted in an overexpression of MALAT1^28^. Taken together, lncRNA polymorphisms could induce the occurrence of tumor by binding to transcription factors, binding to miRNA and so on.

To the best of our knowledge, our research focused on the possible association of lncRNA rs944289 and rs7990916 polymorphisms with CRC risk for the first time. There still existed several limitations in the study. Firstly, this case–control study only focused on Chinese Han populations. Secondly, the two lncRNAs selected in our study may not be comprehensive because there may be other genetic polymorphisms that affected the risk of CRC. Thirdly, we did not perform the association of lncRNA rs944289 and rs7990916 polymorphisms with tumor stages, patients’ prognosis, or other associated risk factors, which might affect the precision of estimating the effect of these two polymorphisms on the CRC risk. Finally, we did not investigate the mechanism of the two polymorphisms during the tumorigenesis of CRC. Nevertheless, our study provides clews for a further mechanism study and provides possible biomarkers for CRC diagnosis.

## Conclusions

In summary, we proved that the lncRNA rs944289 might be significantly related to the decreased risk of CRC in the Chinese Han populations. However, there was no significant difference between lncRNA rs7990916 polymorphism and CRC risk except for the subgroup of age < 61. In the future studies, larger samples including detailed clinical information and different ethnic populations should be conducted to validate our findings.

## Data Availability

All data analyzed or generated during this study are included in the article.
